# Associations of Left Atrial Volume Index to Left Ventricular Ejection Fraction Ratio with Clinical Outcomes in Transthyretin Cardiac Amyloidosis †

**DOI:** 10.3390/jcdd11110363

**Published:** 2024-11-08

**Authors:** Yeabsra K. Aleligne, Machelle D. Wilson, Martin Cadeiras, Michael Gibson, Shirin Jimenez, Stella Yala, Pablo E. Acevedo, David A. Liem, Julie T. Bidwell, Imo A. Ebong

**Affiliations:** 1 Department of Internal Medicine, University of California Davis Health, Sacramento, CA 95817, USA; yka@ad3.ucdavis.edu; 2 Department of Public Health Sciences, Division of Biostatistics, University of California Davis, Sacramento, CA 95616, USA; mdwilson@ucdavis.edu; 3 Division of Cardiovascular Medicine, University of California Davis Health, Sacramento, CA 95817, USA; mcadeiras@ucdavis.edu (M.C.); syjimenez@ucdavis.edu (S.J.); syala@ucdavis.edu (S.Y.); peacevedo@ucdavis.edu (P.E.A.); daliem@ucdavis.edu (D.A.L.); 4 Sutter Medical Foundation, Sacramento, CA 95816, USA; michael.gibson@sutterhealth.org; 5 Betty Irene Moore School of Nursing, Family Caregiving Institute, University of California Davis, Sacramento, CA 95817, USA; jtbidwell@ucdavis.edu

**Keywords:** cardiac amyloidosis, transthyretin, clinical outcomes, echocardiography

## Abstract

Background: Transthyretin amyloid cardiomyopathy (ATTR-CM) affects all cardiac chambers to cause left ventricular (LV) deformation as well as left atrial (LA) remodeling and functional impairment. We investigated the associations of the LA volume index (LAVI):LV ejection fraction (LVEF) ratio with the increased risk of death, heart transplant, or LV assist device implantation (LVAD) in patients with ATTR-CM. Methods: This was a retrospective cohort study involving 69 heart failure (HF) patients with ATTR-CM at an academic medical center between 1 November 2008 and 31 March 2024. ATTR-CM was diagnosed using a technetium–diphosphonate/pyrophosphate scan or an endomyocardial biopsy. The LAVI and LVEF were measured by echocardiography. Cox proportional hazards models were used for the analysis. Results: The mean (SD) age of the participants was 77.5 (9.3) years. Over a median (IQR) follow-up period of 1.96 (0.67–2.82) years, we observed 24 composite events that included twenty-two deaths, two heart transplants, and two LVAD implantations (who subsequently died). In multivariable-adjusted analyses that accounted for age and the glomerular filtration rate, a one-unit increase in the LAVI:LVEF ratio was associated with a doubling of the risk (HR, 95% CI: 2.06, 1.11–3.82) of experiencing the composite outcome. Conclusions: A one-unit increase in the LAVI:LVEF ratio was associated with an increased risk of death, heart transplant, or LVAD implantation in patients with ATTR-CM.

## 1. Introduction

Transthyretin amyloid cardiomyopathy (ATTR-CM) is an infiltrative cardiomyopathy caused by the deposition of transthyretin-derived insoluble amyloid fibrils in the myocardium [1]. Echocardiography is often the initial cardiac imaging modality used to investigate suspected cases of ATTR-CM and typically shows thickened walls, bi-atrial enlargement, a normal or decreased left ventricular (LV) cavity size, diastolic dysfunction, and, at advanced stages, a severe restrictive filling pattern and depressed ejection fraction (EF) [2]. Although a tissue biopsy is the gold standard for diagnoses, a moderate or severe degree of uptake on a nuclear medicine technetium–diphosphonate/pyrophosphate (^99m^TC-PYP) scan and the absence of monoclonal proteins confers a 100% predictive value for ATTR-CM [2]. While the hereditary variant is a consequence of over 100 possible point mutations in the TTR gene, the wild type commonly affects the hearts of older men [3].

Because individuals with ATTR-CM are predisposed to developing disabling cardiac conduction abnormalities, arrhythmias such as atrial fibrillation, and clinical heart failure (HF) [4], it is important to identify patients who are at risk of experiencing adverse outcomes and who require an intensified combination of disease-modifying agents and HF therapies to alter the subsequent progression of their disease. Amyloid fibrils are cytotoxic and initiate a cascade within the cardiac myocytes that leads to myocardial interstitial expansion and contractile dysfunction in both the atrial and ventricular chambers [3,5,6]. This leads to LV deformation [3] as well as LA remodeling, which is a consequence of both an increased wall stiffness from the accumulation of insoluble amyloid fibrils in the atria [6] and a restrictive LV physiology, which impairs the compliance of the LV, thereby generating elevated left-sided filling pressures [3] and, eventually, LV systolic dysfunction in later stages of the disease [7].

Consequently, a modality that concurrently accounts for both LA and LV pathology may be useful for predicting clinical outcomes in ATTR-CM. The LA volume index (LAVI) is a strong predictor of morbidity and mortality in multiple cardiovascular disorders [8]. Likewise, a depressed LVEF is a strong predictor of cardiovascular morbidity and mortality in HF patients [9]. In patients with ATTR-CM who often have a preserved LVEF until very late in their disease [2], the isolated use of the LVEF may be less accurate for appropriately predicting the clinical outcomes. Several clinical indices, including imaging parameters, have been used for monitoring and prognostication in patients with ATTTR-CM [10]. In patients with ST-elevation myocardial infarction (STEMI), a combination parameter, the LAVI:LVEF ratio, was shown to significantly predict major adverse cardiovascular events [11]. Therefore, we investigated the associations of the LAVI when considered in the context of the LVEF (LAVI:LVEF ratio), with important clinical endpoints (the risk of death, heart transplant, or LV assist device [LVAD] implantation) in ATTR-CM. We hypothesized that patients with a greater LAVI:LVEF would have higher risks of death, heart transplant, or LVAD implantation (composite endpoint) as compared to those with lower ratios.

## 2. Materials and Methods

This was a retrospective, chart-based study involving 69 HF patients with a clinical diagnosis of ATTR-CM who received care at the University of California Davis Medical Center (UCDMC), Sacramento in Northern California, USA between 1 November 2008 and 31 March 2024. Approval for this study was obtained from the University of California Davis Institutional Review Board (IRB), Sacramento, CA, USA (IRB ID number 1743084-1). Because it was not feasible to contact the relatives of all the deceased patients, a health insurance portability and accountability act (HIPAA) waiver of consent was granted by the IRB for this study. The time point defined as the baseline for our analysis corresponded to the date when the diagnosis of ATTR-CM was made.

### 2.1. Diagnosis of Clinical Heart Failure

Clinical HF patients were identified based on a physician diagnosis and the use of HF medications, as well as the presence of elevated natriuretic peptides; evidence of pulmonary congestion on chest radiography; or the presence of a decreased LVEF, dilated LV, or LV diastolic dysfunction on echocardiography or cardiac magnetic resonance imaging (MRI).

### 2.2. Diagnosis of Transthyretin Amyloid Cardiomyopathy

HF patients with suspicious findings on cardiac imaging, which included echocardiographic or cardiac MRI evidence of LV hypertrophy (wall thickness ≥ 12 mm), the presence of diffuse late gadolinium enhancement, an elevated extracellular volume, or a native T1 mapping time and an abnormal myocardial nulling pattern on the cine inversion recovery sequences on cardiac MRI, as well as a normal or low voltage on their electrocardiogram and suggestive symptoms of systemic amyloidosis, were further evaluated for ATTR-CM. These symptoms included a history of orthostatic hypotension, carpal tunnel syndrome, trigger finger, spinal stenosis, numbness or tingling in the hands and feet, an unprovoked biceps tendon rupture, and digestive disturbances [12]. The initial evaluation consisted of serological testing with serum immunofixation, urine immunofixation, and a free light chain ratio. Patients with negative serological testing were subsequently evaluated with a ^99m^TC-PYP scan. An endomyocardial biopsy (EMB) was pursued in patients with an equivocal ^99m^TC-PYP scan who had a high clinical suspicion of ATTR-CM. ATTR-CM was confirmed in participants with negative serologic testing based on a positive technetium pyrophosphate scintigraphy (^99m^TC-PYP scan) or amyloid deposits detected on EMB specimens. The use of a ^99m^TC-PYP scan for diagnosing ATTR-CM involves the intravenous administration of 10 to 25 mCi of a radiotracer followed by planar and single-photon emission computed tomography imaging at 3 h [13]. We utilized both a qualitative grading system, the American Society of Nuclear Cardiology (ASNC) score, and a semiquantitative assessment (heart-to-contralateral ratio) to characterize the patients. An ASNC score ≥ 2 and a heart-to-contralateral ratio ≥ 1.3 at 3 h was considered diagnostic for ATTR-CM. Histologic evidence of amyloid deposits in EMB specimens of our patients was indicated by the presence of apple-green birefringence under polarized light after Congo red staining. All positive EMB specimens were sent to the Mayo Clinic laboratories, MN, USA for amyloid typing by mass spectrometry. All patients with ATTR-CM also underwent genetic testing to confirm the amyloid subtype. We included HF patients with either wild-type or hereditary ATTR-CM.

### 2.3. Baseline Measurements

The baseline measurements were obtained at the time of diagnosis of ATTR-CM with a 99mTC-PYP scan or EMB during inpatient hospitalization or in the outpatient clinic. Information on the patients’ age; biological sex; race; height; weight; New York Heart Association (NYHA) functional class; comorbidities, including coronary artery disease (CAD), diabetes mellitus, atrial fibrillation, and hypertension; laboratory parameters, such as the serum creatinine, high-sensitivity troponin T (hs-TnT), and natriuretic peptides (B-type natriuretic peptide and NT-pro B-Type natriuretic peptide); and cardiac implantable electrical devices, such as implantable cardioverter-defibrillators and pacemakers, were obtained by chart abstraction from the electronic medical records. Weight and height were measured with a balance scale and stadiometer, respectively, using standardized clinical protocols. The body mass index was calculated as the weight divided by the square of the height (kg/m^2^). The glomerular filtration rate (eGFR) was estimated from the serum creatinine using the 2021 chronic kidney disease epidemiology collaboration equation. A single measurement of resting blood pressure was obtained using an automated sphygmomanometer in the seated position by trained clinical personnel. Blood samples were collected by trained phlebotomists and analyzed for cardiac troponin and natriuretic peptide levels using standardized laboratory protocols.

### 2.4. Echocardiographic Parameters

Echocardiographic studies were performed by certified sonographers using a GE echocardiography machine and standardized protocols, which were based on guidelines from the American Society of Echocardiography [14]. The LA volume was measured using the biplane area–length method from the apical four- and two-chamber views. The LAVI was calculated by dividing the LA volume by the body surface area. The LVEF was calculated using the modified Simpson’s rule by subtracting the LV end-systolic volume from the LV end-diastolic volume and dividing it by the LV end-diastolic volume. The LAVI:LVEF was calculated by dividing the LAVI by the LVEF. The LV mass index (LVMI) was calculated using the 2-dimensional-based area–length formula [14] and indexed to body size. All echocardiographic measurements were obtained within six months of the time of diagnosis.

### 2.5. Definition of the Clinical Endpoint and Follow-Up

Information on our composite clinical endpoint of death, heart transplants, and LVAD implantation was obtained by chart abstraction of the participants’ medical records. Follow-up for each participant began at the time of the ATTR-CM diagnosis and ended with clinical censoring at the time of the occurrence of any component of the composite clinical endpoint, loss to follow-up, or administrative censoring on 31 March 2024. Two patients experienced both LVAD implantation and death, in which case the time to the first event (LVAD implantation) was used.

### 2.6. Statistical Analysis

The data are presented according to the presence or absence of the composite clinical endpoint using the means ± standard deviation (SD) or the medians (interquartile ranges, IQR) for continuous variables and the percentages for categorical variables, as appropriate. We tested for differences between the groups using Student’s *t*-test and the Kruskal–Wallis test for normal and skewed continuous distributions, respectively, and the chi square test for categorical variables. The participants were censored if they were lost to follow-up or did not experience the composite endpoint at the end of the study. The participants were also categorized as having an elevated LAVI:LVEF ratio if their measurements were greater than the median value. Then, we calculated the incidence rates of death, heart transplant, or LVAD implantation per 100 person-years according to the median split of the LAVI:LVEF ratio. We also plotted Kaplan–Meier curves for death, heart transplant, and LVAD implantation according to the median split of the LAVI:LVEF ratio and tested for differences across the categories with the log rank test. We also present the characteristics of the study participants according to the median split of the LAVI:LVEF ratio.

Cox proportional hazards models were used to investigate the associations of continuous and categorical measures of the LAVI:LVEF ratio separately with the composite outcome. We constructed unadjusted models (model 1) and age-adjusted models (model 2). In model 3, we introduced other clinically relevant variables, such as the biological sex, race, body mass index, type of ATTR-CM, presence of cardiac implantable electrical devices, NYHA functional class, presence of moderate or severe mitral regurgitation, estimated glomerular filtration rate, LVMI, and comorbidities such as hypertension, diabetes mellitus, atrial fibrillation, and CAD. Due to our limited sample size, we selected the most parsimonious model using backward elimination, in which we retained age and eliminated variables if their *p*-value was greater than 0.05. The resulting model for continuous measures of the LAVI:LVEF ratio included age and the glomerular filtration rate, while that for categorical measures of the LAVI:LVEF ratio included age, the glomerular filtration rate, the presence of cardiac implantable electrical devices, and moderate to severe mitral regurgitation. The proportional hazards assumption was tested by a visual examination of the log–log plots. A statistical analysis was performed using the SAS^®^ software, version 9.4, for Windows^®^ (SAS Institute Inc., Cary, NC, USA). *p*-values < 0.05 were considered statistically significant.

## 3. Results

The mean age of the participants was 77.5 (SD +/− 9.3) years. The mean value for the LAVI:LVEF ratio was 1.04 (SD +/− 0.67). Twenty of the participants were diagnosed during inpatient hospitalization, while forty-nine were diagnosed in the outpatient clinic. The median value of the LAVI:LVEF ratio appeared similar, at 0.96 (interquartile range: 0.78–1.36) and 0.84 (interquartile range: 0.59–1.03) for participants who were diagnosed during inpatient hospitalization and in the outpatient clinic, respectively (*p* = 0.09).

**Figure 1:**
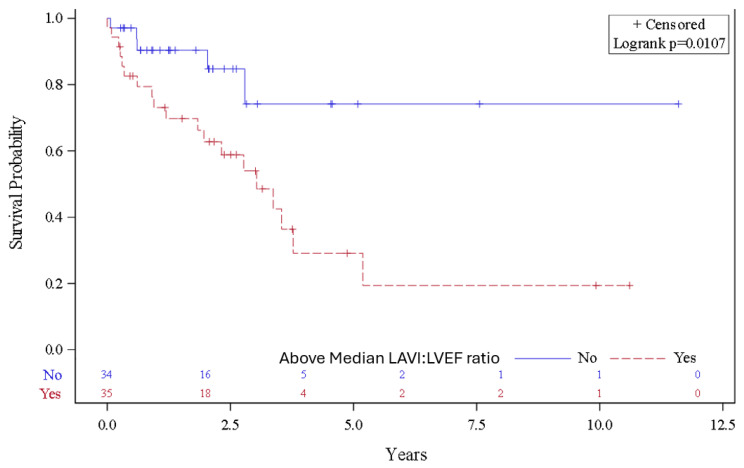
The probability of death, heart transplant, or left ventricular assist device implantation for participants with a left atrial volume index (LAVI)/left ventricular ejection fraction (LVEF) ratio above and below the median value. The median value of the LAVI:LVEF was 0.86.

The majority (88.4% of the participants) had wild-type ATTR-CM. Most cases (71% of the participants) were diagnosed using the ^99m^TC-PYP scan. Over a median (IQR) follow-up period of 1.96 (0.67–2.82) years, we observed twenty-four events in our sample of 69 patients, which included twenty-two deaths, two heart transplants, and two LVAD implantations. The two patients who received LVAD implants subsequently died and were only included once for the purposes of our analyses. The rate of occurrence of the composite outcome was 22.7/100 and 6.7/100 person-years for participants with LAVI:LVEF ratios above and below the median values, respectively. The probability of experiencing the composite outcome at the end of the follow-up was significantly higher among participants with LAVI:LVEF ratios above the median value (Figure 1).

Patients who experienced the composite outcome more commonly had moderate to severe mitral regurgitation, a lower GFR, and a cardiac implantable electrical device at the time of diagnosis, and were more likely to be diagnosed during inpatient hospitalization for HF (Table 1). Patients with a LAVI:LVEF ratio above the median value had a lower GFR (Table 2). In multivariable-adjusted analyses that accounted for age and the GFR, a one-unit increase in the LAVI:LVEF ratio was associated with a doubling of the risk of experiencing the composite outcome (HR, 95% CI: 2.06, 1.11–3.82) (Table 3). In multivariable-adjusted analyses that accounted for age, the GFR, moderate to severe mitral regurgitation, and the presence of cardiac implantable electrical devices, values of LAVI:LVEF above the median value were not associated with an increased risk of experiencing the composite outcome (HR, 95% CI: 1.58, 0.63–3.96) (Table 3).

## 4. Discussion

In this single-center retrospective cohort study of 69 HF patients with ATTR-CM in a tertiary care center, a one-unit increase in the LAVI:LVEF ratio was independently associated with an increased risk of death, heart transplant, or LVAD implantation. Although not previously evaluated in the ATTR-CM population, the LAVI:LVEF ratio is useful for the prediction of MACE in patients with STEMI [11]. When the LAVI:LVEF ratio was dichotomized according to the median cutoff point, values above the median were not independently associated with an increased risk of the composite endpoint. However, our categorical analysis was exploratory in nature, as there are no known clinically relevant cutoff points for the LAVI:LVEF and this may have been limited by our modest sample size.

Atrial enlargement is commonly reported among patients with ATTR-CM [5]. By itself, atrial enlargement is a recognized marker of late cardiac pathology [5], and LA volumes have been linked with morbidity and mortality in various cardiovascular conditions [3,15]. Atrial enlargement is also highly correlated with LV deformation [3], which is not surprising because the LA is both a conduit and an active pump, and significantly contributes to LV filling and the stroke volume [15]. Additionally, if there is increased LV wall thickness, as is often reported among patients with ATTR-CM, the LA myocardial function can be abnormal, irrespective of its size/volume [15]. Thus, the LA is an indirect indicator of LV chamber compliance and diastolic function, as well as the intracardiac pressure and volume overload [11]. When considered in isolation, the LA size may have less prognostic utility in ATTR-CM [3]. It is also important to note that changes in the LA wall structure can cause impairments to its reservoir and pump function, irrespective of the LA volume and LVEF [6].

The LV function is an important echocardiographic index in ATTR-CM. Although ATTR-CM commonly presents with a preserved LVEF, the range of the LVEF at diagnosis is variable [16]. Similar to other HF syndromes [17,18], a lower LVEF indicates a more advanced disease and a worse prognosis [19]. In fact, among patients with a decreased LVEF at presentation, an improvement or stability in LV function has been associated with better clinical outcomes than a decrease in the LVEF over time [20]. In some instances, the LVEF may be reduced even with a normal LV chamber size [16] and is more useful than the LV size/dimensions in predicting clinical outcomes [21]. However, morbidity and mortality are significantly increased when LV dysfunction and LV enlargement (increased in the LV end diastolic dimensions) are simultaneously present [21]. In instances when both the LAVI and the LVEF are within normal limits, a combined parameter may provide an improved risk prediction above the individual indices in isolation [11].

### Strengths and Limitations

This study evaluated the clinical outcomes of ATTR-CM in a real-world setting using echocardiography, which is a commonly available cardiac imaging modality. The study location was an academic medical center, and as such, additional diagnostic modalities such cardiac MRI, a ^99m^TC-PYP scan, and an endomyocardial biopsy were readily available and evaluated by expert personnel, thereby enhancing the confidence in the diagnostic accuracy of ATTR-CM in the study cohort. Our study has high clinical relevance because the prognosis of patients with TTR amyloidosis is determined by the presence and extent of cardiac involvement [22], making it crucial to identify cardiac predictors of adverse clinical outcomes. This study also has limitations. The data was collected from a single center and the sample size was relatively small and consisted predominantly of older male participants. As a result, our findings may not be generalizable to other geographic locations, or to younger or female populations. However, the age and gender distribution seen in our cohort is representative of that in the general population of amyloid patients [23], especially those with wild-type disease. Lead-time bias is also possible because the increased availability of the non-invasive ^99m^TC-PYP scan has enabled earlier diagnoses of the disease, and the use of disease-modifying agents like Tafamidis in recent years will likely lead to improved outcomes and bias our findings to the null. This is particularly relevant for this study because, even though our observations began in November 2008, a large proportion of the cohort (over 50%) were diagnosed in the last 3 years and received Tafamidis during follow up. This matches the diagnostic trends reported in other studies, where there has been a substantial increase in ATTR-CM diagnoses because of the availability of advanced cardiac imaging [24] and decreased mortality due to the detection of the disease at earlier stages [24]. Due to our relatively small sample size, we were unable to further explore associations by TTR subtypes, and caution must be applied to the findings from our categorical analysis. There is the possibility of residual confounding from unmeasured risk factors that were not accounted for in our parsimonious models. Natriuretic peptides were only available in a subset of participants, and this parameter was not included in the multivariable models.

## 5. Conclusions

In the present study, a one-unit increase in the LAVI:LVEF ratio was independently associated with an increased risk of death, heart transplant, or LVAD implantation. This finding should be further explored in adequately powered cohorts. Further studies are needed to determine if there is an incremental role for the LAVI:LVEF ratio as a predictor of clinical outcomes in ATTR-CM beyond the currently available risk-scoring systems.

## Figures and Tables

**Table 1:** Characteristics of the study participants according to the presence or absence of the composite outcome at the end of the follow-up.

Characteristic	Present	Absent	*p*-Value
Age, years	78.4 (10.75)	77.0 (8.48)	0.58
Female sex, %	20.8	13.3	0.42
Non-White race, %	25.0	13.3	0.22
Inpatient diagnosis, %	54.2%	15.6%	0.002
Hereditary transthyretin cardiac amyloidosis, %	12.5	11.1	0.86
New York Heart Association functional class 3 or greater, %	70.8	48.9	0.078
Cardiac implantable electrical device, %	66.7	33.3	0.008
Body mass index, kg/m^2^	26.9 (4.34)	28.0 (5.62)	0.39
Estimated glomerular filtration rate, mL/min/1.73 m^2^	42.5 (35.5–46.0)	52.0 (46.0–63.0)	0.0006
B-type natriuretic peptide ^a^, pg/mL	721.0 (383.0–1039.0)	341.0 (234.0–703.0)	0.053
N-terminal pro-B-type natriuretic peptide ^b^, pg/mL	2263.0 (1337.0–5148.0)	1477.0 (629.0–3657.0)	0.16
High-sensitivity troponin T ^b^, ng/L	75.5 (37.5–154.5)	41.5 (29.5–73.5)	0.065
Hypertension, %	87.5	88.9	0.86
Diabetes mellitus, %	41.7	35.6	0.62
Atrial fibrillation, %	100	86.7	0.06
Coronary artery disease, %	79.2	57.8	0.076
Left atrial volume index, mL/m^2^	45.5 (40.2–61.1)	42.7 (34.1–51.6)	0.14
Left ventricular ejection fraction (%)	40.5 (28.6–55.0)	56.0 (50.0–60.0)	0.0006
Left ventricular mass index, g/m^2^	158.3 (118.6–181.6)	139.0 (102.1–177.9)	0.33
LAVI:LVEF ratio	1.0 (0.9–1.7)	0.8 (0.6–1.0)	0.0006
Moderate/severe mitral regurgitation, %	45.8	13.3	0.0028

The composite outcome consisted of death, heart transplant, or left ventricular assist device implantation. We observed twenty-four events in total, which included twenty-two deaths, two heart transplants, and two LVAD implants (who subsequently died). ^a^ Only 45 participants had B-type natriuretic peptide measurements, ^b^ while 48 participants had N-terminal pro-B-type natriuretic peptide and high-sensitivity troponin T measurements. The values are presented as percentages for categorical variables and the median (interquartile range) for continuous variables. Differences between the groups were analyzed using a chi square test or Fisher’s exact test for categorical variables, and a two-sample Student’s *t*-test or the Kruskal–Wallis test for continuous variables as appropriate. Abbreviations: LAVI:LVEF ratio, left atrial volume index to left ventricular ejection fraction ratio.

**Table 2:** Characteristics of the study participants above and below the median value of the LAVI:LVEF ratio at the time of diagnosis.

Characteristic	Above the Median	Below the Median	*p*-Value
Age, years	78.4 (9.36)	76.6 (9.25)	0.44
Male sex, %	80.0	88.2	0.51
Non-White race, %	22.8	11.8	0.34
Inpatient diagnosis, %	37.1	20.6	0.21
Hereditary transthyretin cardiac amyloidosis, %	8.6	14.7	0.48
New York Heart Association functional class 3 or greater, %	60.0	52.9	0.63
Cardiac implantable electrical device, %	57.1	32.4	0.05
Body mass index, kg/m^2^	27.0 (5.23)	28.2 (5.19)	0.35
Estimated glomerular filtration rate, mL/min/1.73 m^2^	44.4 (15.9)	54.6 (14.8)	0.007
B-type natriuretic peptide ^a^, pg/mL	548.5 (319.0, 874.0)	391.0 (252.0, 924.0)	0.70
N-terminal pro-B-type natriuretic peptide ^b^, pg/mL	1731.0 (1253.0, 4332.0)	1233.0 (374.0, 3657.0)	0.13
High-sensitivity troponin T ^b^, ng/L	62.0 (38.0, 124.0)	40.0 (30.0, 48.0)	0.48
Hypertension, %	85.7	91.2	0.71
Diabetes mellitus, %	34.3	41.2	0.62
Atrial fibrillation, %	100	95.2	0.01
Coronary artery disease, %	68.6	61.8	0.62
Left atrial volume index, mL/m^2^	55.7 (15.8)	36.5 (10.1)	<0.0001
Left ventricular ejection fraction, %	42.8 (13.7)	58.9 (6.56)	<0.0001
Left ventricular mass index, g/m^2^	162.7 (56.6)	127.9 (41.8)	0.005
Moderate/severe mitral regurgitation, %	34.3	17.2	0.09

The median value of the LAVI:LVEF ratio was 0.86. ^a^ Only 45 participants had B-type natriuretic peptide measurements, ^b^ while 48 participants had N-terminal pro-B-type natriuretic peptide and high-sensitivity troponin T measurements. The values are presented as percentages for categorical variables and the mean (standard deviation) or median ^a,b^ (interquartile range) for continuous variables. Differences between the groups were analyzed using the chi square test or Fisher’s exact test for categorical variables, and a two-sample Student’s *t*-test or the Kruskal–Wallis test for continuous variables, as appropriate. Abbreviations: LAVI:LVEF ratio, left atrial volume index to left ventricular ejection fraction ratio.

**Table 3:** Association of continuous and categorical measures of the LAVI:LVEF ratio with the composite outcome.

	LAVI:LVEF Ratio		Elevated LAVI:LVEF Ratio	
	HR (95% CI)	*p*-Value	HR (95% CI)	*p*-Value
Model 1	2.70 (1.63–4.47)	0.0001	3.36 (1.25–9.03)	0.02
Model 2	2.65 (1.57–4.46)	0.0002	3.25 (1.20–8.75)	0.02
Model 3	2.06 (1.11–3.82) ^a^	0.02	1.58 (0.63–3.96) ^b^	0.41

The composite outcome consisted of death, heart transplant, or left ventricular assist device implantation. The LAVI:LVEF ratio was categorized as elevated when it was greater than the median value. The median value of the LAVI:LVEF ratio was 0.86. We observed twenty-four events in total, which included twenty-two deaths, two heart transplants, and two LVAD implants (who subsequently died). Model 1, unadjusted analysis. Model 2, age-adjusted analysis. Model 3 ^a^, model 2 adjusted for age and the glomerular filtration rate. Model 3 ^b^, model 2 adjusted for age, the glomerular filtration rate, the presence of cardiac implantable electrical devices, and moderate to severe mitral regurgitation. The hazard ratios were estimated using Cox proportional hazards models and calculated per unit increase in the LAVI:LVEF ratio. Variable selection was performed using backward elimination, in which age was retained and variables were eliminated if their *p* > 0.05. N-terminal pro-B-type natriuretic peptide and high-sensitivity troponin T measurements were available for less than 70% of the participants and were not included in the variable selection process. Abbreviations: LAVI:LVEF ratio, left atrial volume index to left ventricular ejection fraction ratio.

## Data Availability

Due to privacy, ethical restrictions, and ongoing collection of data, the data used in this study is unavailable for sharing.
